# Variation in Indigenous Forest Resource Use in Central Guyana

**DOI:** 10.1371/journal.pone.0102952

**Published:** 2014-07-28

**Authors:** Claire M. P. Ozanne, Christie Cabral, Peter J. Shaw

**Affiliations:** 1 Centre for Research in Ecology, Department of Life Sciences, University of Roehampton, London, United Kingdom; 2 School for Social & Community Medicine, University of Bristol, Bristol, United Kingdom; New York State Museum, United States of America

## Abstract

Sustainable forest conservation strategies should be based on local as well as landscape-scale forest resource use data. Using ecological and sociological techniques, we test the hypotheses that (1) forest resource use differs between ethnic and socioeconomic indigenous groups and (2) that this difference results in differing spatial patterns of resource use, with implications for forest diversity and for conservation planning. In the North Rupununi Guyana, three adjacent indigenous communities (differing in their indigenous/immigrant balance) were recorded using 73 animal and 164 plant species (plus several unidentified ethno-species). Farm sites formed important foci for most forest based activities and ex-farm sites supported similar floristic diversity to surrounding forest. Resource usage differences between communities could be attributed to socio-cultural drivers, e.g. mammal meat consumption and the use of the fruits from the palm tree *A. maripa* were higher in more traditional households. When extracting household construction timber, lower income groups created small scattered felling sites akin to tree fall gaps whereas higher income groups created larger gaps. Lower income (indigenous) households tended to clear larger but more contained sites for farming while mixed or non-Amerindian household tended to clear smaller but more widely dispersed farm sites. These variations resulted in different patterns of forest disturbance originating from agriculture and timber extraction.

## Introduction

Global strategies to reduce the rate of forest loss include setting up Protected Areas (PAs) of various types (including those which prohibit exploitation and those which allow it), the designation of Indigenous Reserves (IRs) and of Community Managed Forests. Recent evaluation of the effectiveness of IRs in the Amazon indicates that they play a significant role in reducing the number of deforestation fires [1]. In the Colombian Guyana Shield both Protected Areas and Indigenous Reserves act as buffers to deforestation [2]), particularly where large scale agriculture is a key agent of change [3] and recent global analyses of CMFs indicate they can be effective at reducing deforestation in the tropics [4]. Whilst gazetting land clearly has a positive impact overall [4] there is considerable variation in the effectiveness at a local level. Differences in the degree of protection afforded by the designated areas are dependent on factors such as road proximity and pattern and practices of colonisation [5], with density of habitation and approaches to management and resource use being key [1], [2], [3], [6]. Much tropical forest lies outside protected areas and there remains a lack of information about anthropogenic effects on biodiversity patterns in these areas which is needed to inform regional conservation plans [8]). Although higher order analysis of land use change is of importance in the development of conservation strategies and in predicting impact of climate change, if models are to respond usefully to the complexity of this process, local “place based” information is required [9], [10], [11]. Such inputs should encompass both historically traditional and emergent patterns of management and resource use and must involve data collection at both ecologically and sociologically relevant scales.

Research in the Brazilian Amazon [12], Mexico [13], South Korea [14] and Tanzania [10] has documented the interdependence of indigenous people and forest habitat, as well as the influence of this interaction on the spatial arrangement of a wide range of timber and non-timber forest (NTFPs) products and resources. Recent studies have focussed on spatial patterns of hunting [7], [15] and the effects on vertebrate conservation work. It is apparent from these studies that a good understanding of the effects of resource use requires disentangling the differing patterns across particular ethnic and socioeconomic groups within communities [6].

The Guiana shield, encompassing parts of northern Brazil, French Guiana, Suriname, Venezuela and eastern Colombia, supports the largest unfragmented extent of ‘frontier forest’ in the world [16]. In Guyana approximately 80 percent of the land remains forested, with high levels of endemism [17], [18]. Of this forest, approximately 80 percent is categorised as State owned and 13.9 percent held by the indigenous Amerindian communities [19]. The main threats to the forest are logging, gold mining and the Georgetown to Lethem road. However, the deforestation rate remains relatively low, standing at approximately 0.3 percent per annum (543 ha/annum) [20] (0.29% in 2009) [21].

Local communities are integral to the protection of these areas as well as those owned by the state and the forest that does not lie within the protected zones and yet many remain dependent on forest resources for their livelihoods.

The objectives of this study were to test the hypotheses that: (1) forest resource use (animal and plant) varies between ethnic and socioeconomic indigenous groups and (2) that this variation results in differing spatial patterns of resources use.

### Study area

**Figure 1:**
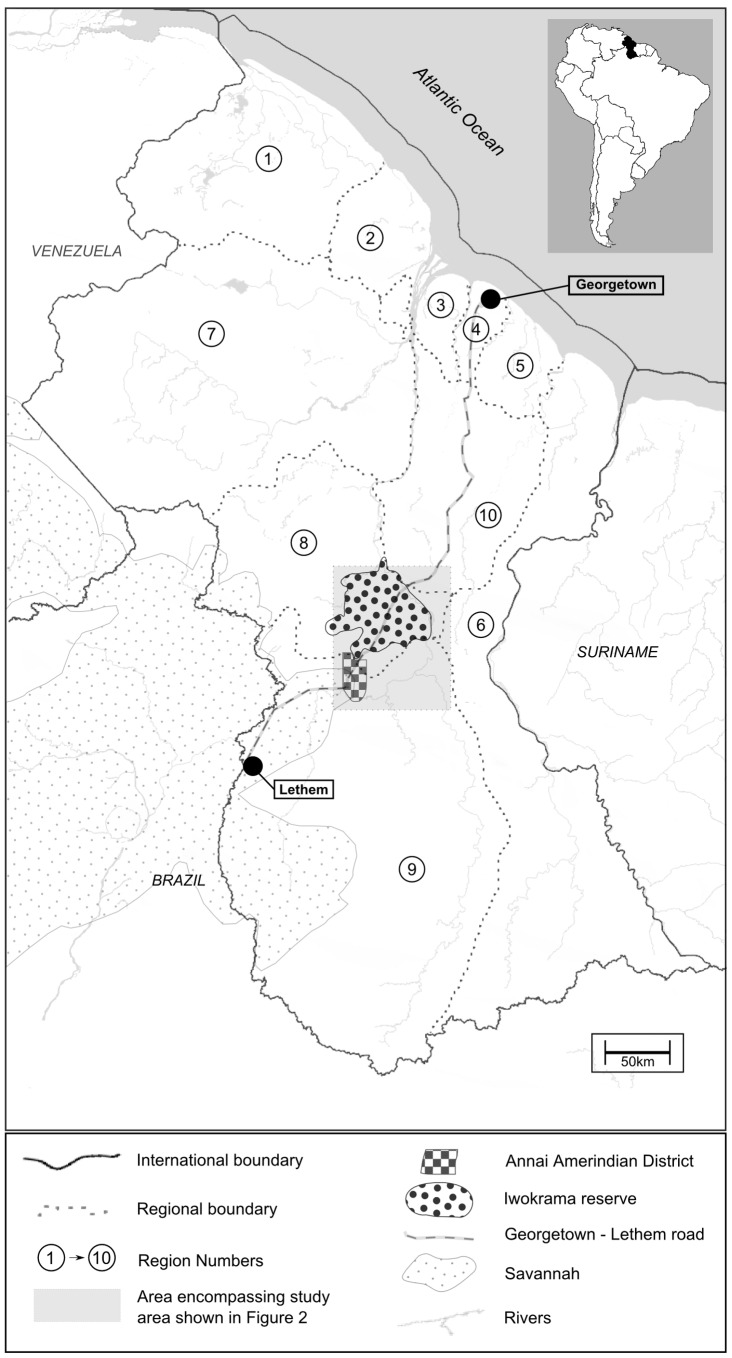
Map showing location of study site in central Guyana, South America [67].

**Figure 2:**
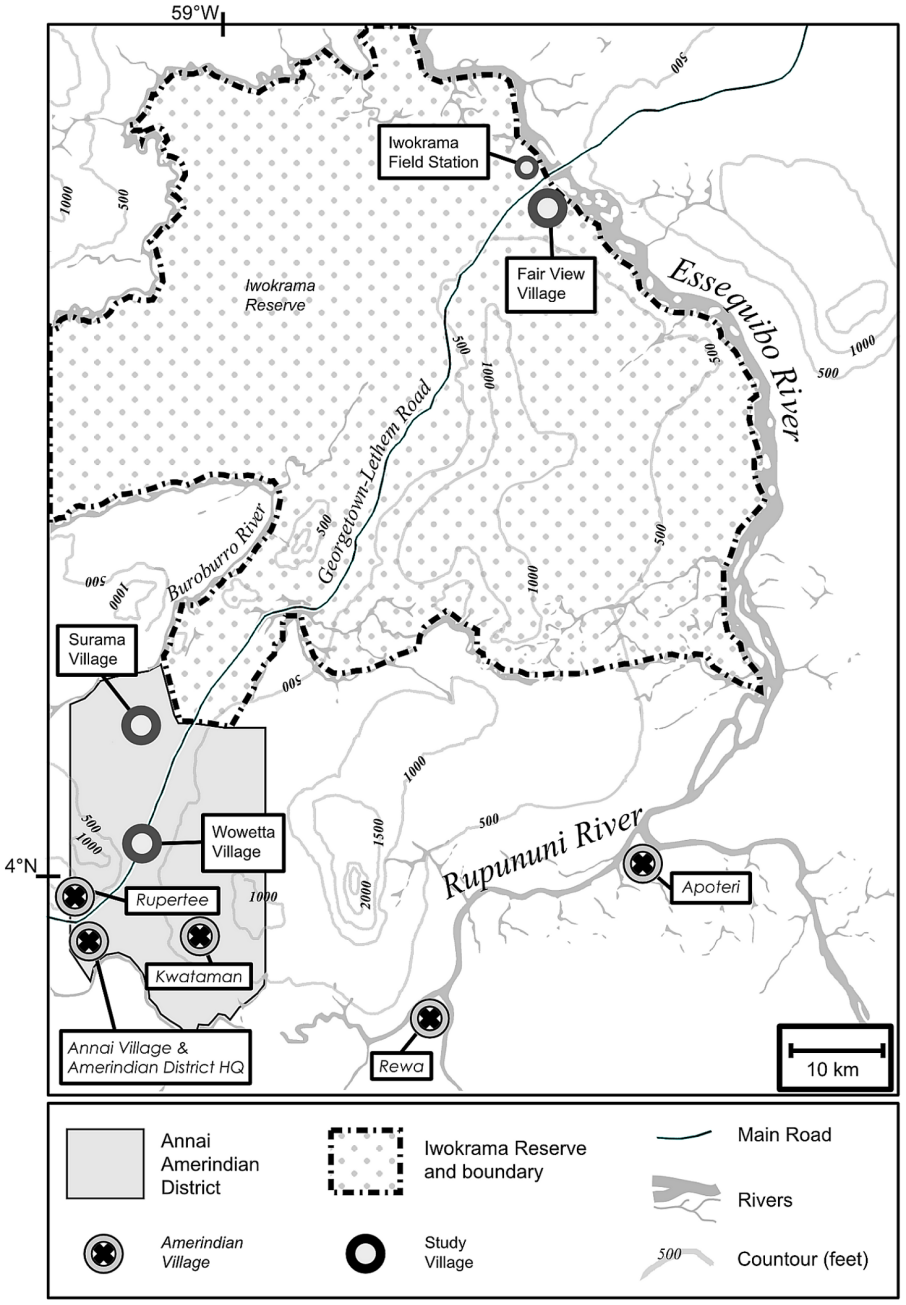
Map showing location of study villages in the Rupununi, Guyana [68].

Our study was conducted in central Guyana (4° 20′N, 59°10′W, see Figure 1) where the North Rupununi Savanah meets the southern border of the Iwokrama Wilderness Reserve. We worked with three Amerindian communities: Fair View (4°39′ N; 58° 41′ W), the only village located within the Iwokrama Reserve, and Surama (4° 8′ N; 59° 4′ W) and Wowetta (4° 1′ N; 59° 4′ W), two savannah based villages within the Annai Amerindian District which were closest to the Iwokrama Reserve boundary (Figure 2).

The study area is roughly encompassed by the Essequibo, Rupununi and Burroburro rivers [22]. There are two distinct rainy seasons: a long one from May to mid-August (which creates seasonal swamps; [23] and a short one from November to January. In the north, Fair View is surrounded by mixed forest with some nearby patches of ‘muri-scrub’, a degraded form of a fire prone forest type that occurs when fires are very regular [24], [25]. Wowetta is just within the northern boundary of the savannah, with mixed forest all around, while the Surama is in an area touched by several types of mixed forest with a very small area of savannah.

## Materials and Methods

Informed consent was obtained from all participants. Where possible written consent was obtained; however, many of the participants were not sufficiently literate to be able to provide written consent, in these cases verbal consent was obtained and recorded by the researcher. All procedures were approved by the University of Roehampton Committee on Ethics.

Permits to carry out this work were obtained from the Ministry of Amerindian Affairs and from the Environmental Protection Agency in Guyana. All necessary permits were obtained for the described study, which complied with all relevant regulations.

### Participants

The majority of Amerindians living in the North Rupununi belong to the Makushi tribe [26]. At the time of our research, 94 percent of the population of the study communities were Amerindian, mainly of the Makushi tribe (62%), with some from the Arawak (3%) and Wapishana (7%) tribes, and some mixed-tribe households (22%). The residents of Surama and Wowetta were both predominantly Makushi, while in Fair View these three tribal groups were fairly equally represented and there was a higher proportion of mixed-tribe households. A further 5 percent were of mixed-ethnicity (one non-Amerindian parent) and there were two (<1%) residents of non-Amerindian ethnicity both living with Amerindian partners (one in Surama and one in Fair View). We grouped all 84 households (defined as a group of people living in a discrete compound who communally gather, manage and use forest resources) into three classes: Makushi in which both household heads (male and female) were Makushi; Mixed tribe in which both household heads were Amerindian but not both Makushi; Mixed ethnicity in which at least one head is either of non-Amerindian ethnicity or of mixed ethnicity.

All households engaged to varying degrees in both traditional subsistence farming, hunting and gathering practices and in trading or work for cash payment. Study participants ranked household income and we confirmed ranks by visual observation of household possessions; the rankings were then combined and simplified into four roughly equally spaced income groups, 1 being the lowest income group and 4 being the highest.

### Data collection methods

In researching resource use practices, we used a combination of participant observation [27], [28] semi-structured interviews [29], [30] and elicitation techniques from participatory methodologies such as free listing, ranking (used during interviews) and transect walking and mapping (used during participant observation) [27], [28], [29], [31]. We carried out interviews in English or Makushi, the latter using local translators.

Data were collected for two periods; five months in 1998 and 13 months from early 1999–2000. Data on normal patterns of resource use were collected during the first period. Semi-structured interviews with 48 key informants elicited descriptions of hunting, fishing, harvesting of wild plant products and farming practices covering topics including seasonality, locations and reasons behind resource use decisions. Descriptions of these activities were confirmed and further explored through participant observations (following the methods of [32], [33]) during which the researcher joined participants during resource use activities. During the second period, a sample of 43 households (roughly half the households in each village, attempting to sample equally across income and ethnicity) was used to quantify resource use. In Surama, householders were asked to keep resource use diaries recording all plant and animal resources used during the month and the researcher visited each household monthly to collect the diaries and to conduct semi-structured interviews about their resource use practices. Topics covered in the semi-structured interviews included: what resources had been collected, in what quantities, from which locations, to which household resources had been distributed and why those resource use decisions had been taken. Where possible, quantities were measured using hand held scales (for weights) or visual counts (for individual items). However, where part of the resources had already been consumed, participants were asked to estimate the quantities of the original amount harvested. The accuracy of participant weight estimation was measured and average error rates were found to be <5%, with no bias toward either over or under estimation. Sample households recorded resources used for 52 consecutive weeks from March 1999. In Wowetta and Fair View, the researcher visited monthly and participating households were asked to recall and report resource use from the previous week. These two villages were visited ten times at 4–6 week intervals during the same period from March 1999. The quantities of resources recorded for each household were scaled according to the number and composition of each household using standard consumption units (C.U). Standard consumption units are scaled relative to adult male consumption (which scores 1) and are calculated for each gender in several age bands [34]. Seasonal differences were compared by looking at consumption during the two wet seasons (June to Sept; Dec) and the two dry seasons (Oct-Nov; Jan-May).

Forest composition surveys involved locating quadrats (0.25ha) in a stratified random design, sampling 4 different forest types (palm forest: 12 plots, mixed forest: 27 plots, Mora forest: 15 plots, mixed mora: 7 plots) and ex-farm sites: 15 plots aged approximately 5, 10, 15, 20 and 30 years (3 replicates per age). The sampling method follow that of Johnston and Gillman [35] and Johnston [36] for surveying locally defined forest types: local guides located areas they deemed typical of a local forest type and identified the ethno-species within them. This process was repeated in the major forested areas around the community so plots for a certain forest type were drawn from several locations, all within 5 km of the community. Trees over 10 cm dbh were identified to species using [37]–[45]. A herbarium reference collection was prepared in duplicate and one set of specimens were deposited at the University of Guyana Herbarium (BRG<IH>) where field identification were confirmed by the curator of the botanical collection. (See S1 for list of deposited specimens).

Statistical analyses used ANOVA where data met the assumptions or could be transformed to meet them; otherwise non-parametric tests (Kruskal-Wallis) were used [46]. When multiple significance tests were carried out on the same data, a new α value, appropriate for the number of tests carried out, was calculated using the Dunn-Šidák method as described in [47]. Diversity was calculated using Simpson's index: D = 1 – Σp_i_^2^ [48] (using proportional composition of all 78 tree species), and ordinated using PCA on log(X+1)-transformed data for species whose mean density per plot exceeded 1.0 (the commonest 15 species) [48].

## Results

### Household profiling

A breakdown of the villages by income level and ethnicity is given in Table 1. The demographic profiles of these communities are similar to other Amerindian communities in Amazonia, although having more in common with relatively acculturated groups.

**Table 1:** Number of households in each village, income and ethnicity class in a study of the North Rupununi, Guyana.

	Income class	Ethnicity class			
Village		1	2	3	Total
Surama	1	0 [1]	[0]	[0]	**0 [1]**
	2	3 [8]	0 [1]	[0]	**3 [9]**
	3	4 [6]	2 [5]	[0]	**6 [11]**
	4	0 [1]	6 [9]	2 [3]	**8 [13]**
	**Total**	**7 [16]**	**8 [15]**	**2[3]**	**17 [34]**
Fair View	1	[0]	[1]	1 [1]	**1 [2]**
	2	[0]	6 [9]	[1]	**6 [10]**
	3	[0]	2 [4]	1 [2]	**3 [6]**
	4	[0]	[0]	[0]	
	**Total**		**8 [14]**	**2 [4]**	**10 [18]**
Wowetta	1	2 [5]	1 [1]	[0]	**3 [6]**
	2	7 [13]	3 [8]	[0]	**10 [21]**
	3	2 [2]	1 [3]	[0]	**3 [5]**
	4	[0]	[0]	[0]	
	**Total**	**11 [20]**	**5 [12]**	**[0]**	**16 [32]**

(Total number of households in each class is given in square brackets): Ethnicity classes: 1 =  makushi, 2 =  Mixed amerindian, 3 =  Mixed ethnicity.

### Forest types

**Figure 3:**
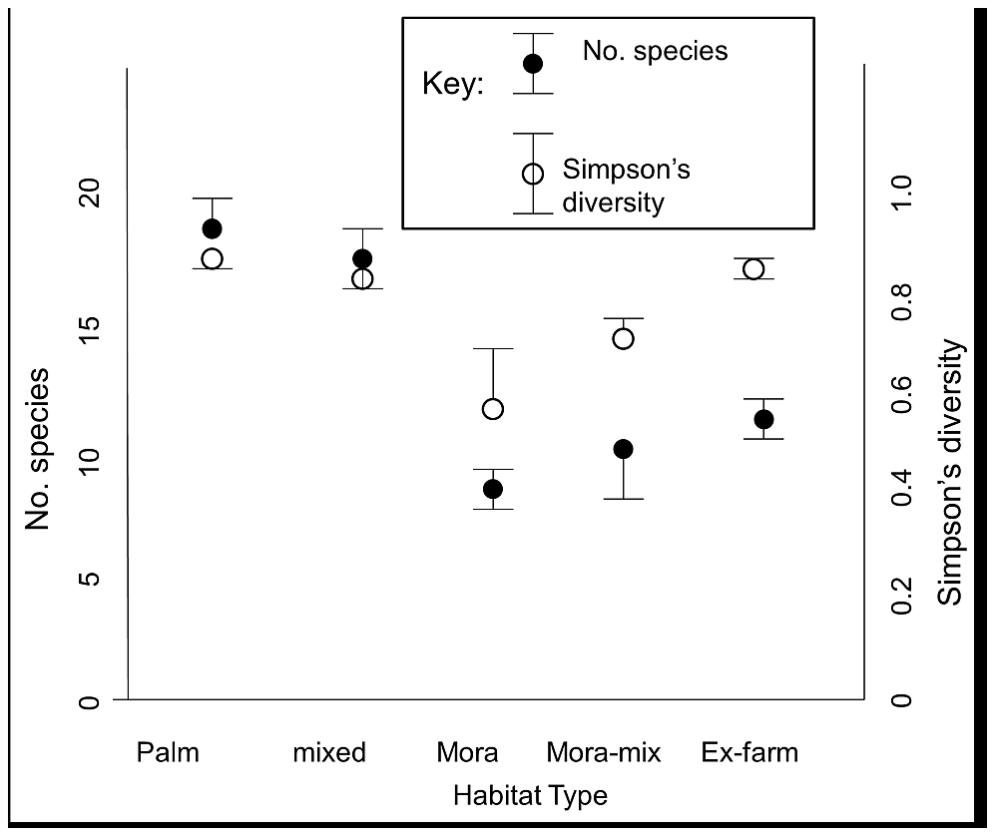
Floristic diversity and species richness in 4 forest types including ex-farm plots, North Rupununi, Guyana. (Representing the 15 dominant species).

**Figure 4:**
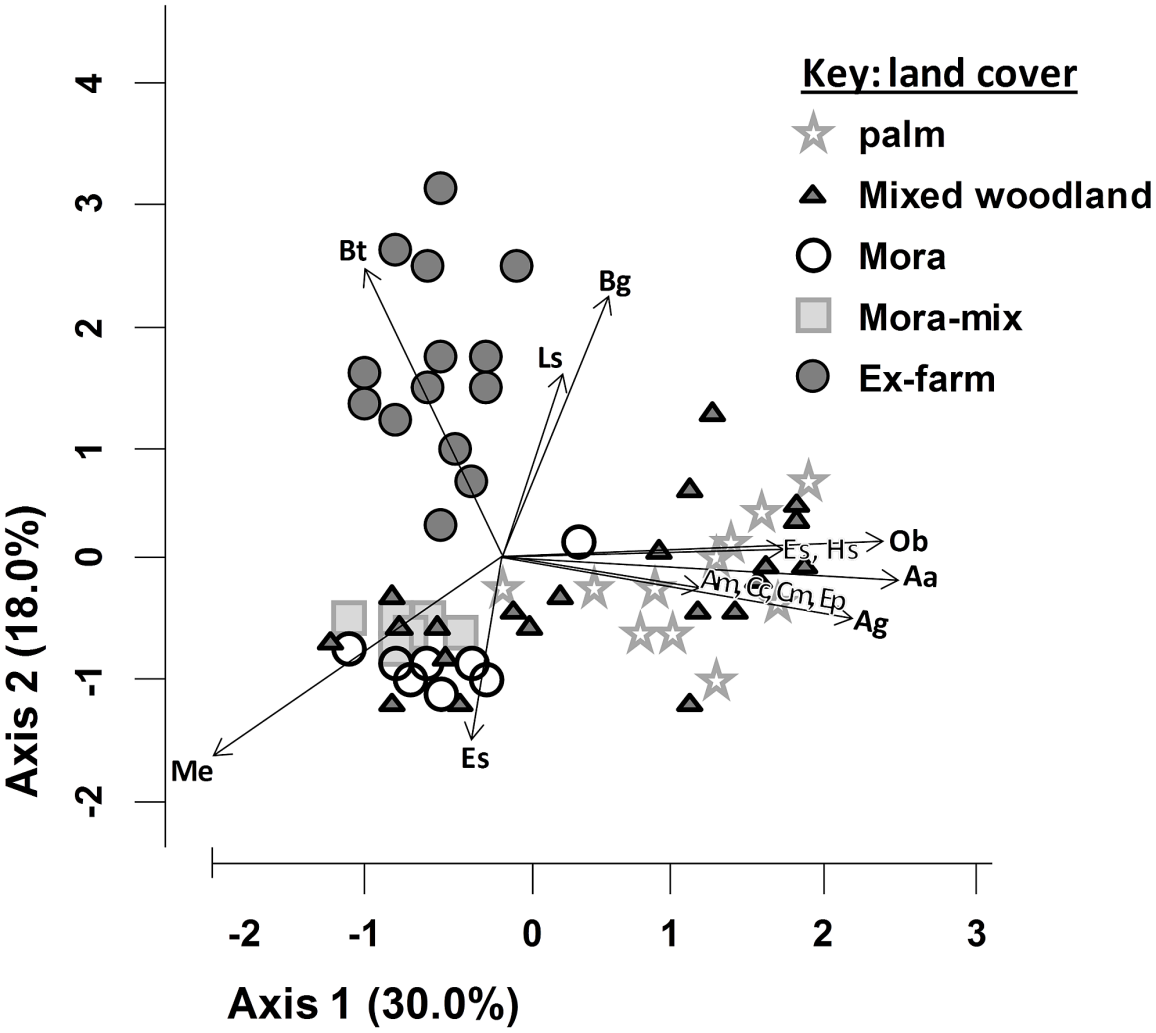
PCA ordination of floristic data (15 dominant tree species) for 4 forest types including ex-farm plots, North Rupununi Guyana. Key: Aa: *A. aculeatum*, Ag: *A. gynacanthum*, Am: *A. maripa*, Bg: *B. guianensis*, Bt: *B. stipulacea*, Cc: *C. commune*, Cm: *C. multiflora*, Es: *E. subglandulosa*, Es: *E. subglandulosa*, Hs: *H. spathocircinata*, Ls: *L. sapotis*, Me: *M. excels*, Ob: *O. bacaba*.

A total of 78 tree species were recorded in the forest plots, of which the dominant 15 (mean value >1 tree per plot) were used in floristic analyses and are listed in Table 2. The ex-farm plots had slightly lower total numbers of species than “Palm” and “Mixed” forest types, but were dominated by scarcer species leading to comparable diversity (Figure 3). PCA ordination of the tree data showed clear clustering (Figure 4). Mora and Mora-mix stands clustered together (and were associated with *Mora excelsa* Benth, unsurprisingly). Palm forest contained the greatest number of common species (notably *Attalea maripa* (Aubl.) Mart, *Astrocaryum aculeatum* G.Mey and *Couratari multiflorum* Eyma). Ex-farm plots also contained a distinct community dominated by *Byrsonima stipulacea* Juss, along with *Loxopterygium sagotti* Hook. When all non-farm sites were excluded, there were no significant differences between the tree communities in different ages of ex-farm plots (Kruskal-Wallis test with 4df; all p>0.05).

**Table 2:** Interquartile range for the dominant 15 tree species) for 4 forest types and farm plots in the North Rupununi, Guyana.

Species	Forest type				
	Palm	Mora	Mora/mixed	Mixed	Farm plots
*Attalea maripa* (Aubl.) Mart.	15–33	0–10	8–12	4–17	3–12
*Mora excels* Benth.	0	17–26	21–25	0–3	0
*Astrocaryum gynacanthum* Mart	6–13	0–5	0–5	0–11	0
*Astrocaryum aculiatum* G.Mey	3–8	0	0	0–7	0
*Oenocarpus bacaba* Mart.	0–3	0	0	0–10	0
*Eschweilera subglandulosa* (Steud. ex O.Berg) Miers	0–4	0–6	0–1	0–4	0
*Heliconia spathocircinata* Aristeg.	0–3	0	0	0–2	0
*Catostemma commune* Sandwith	1–6	0–1	0–1	1–6	0
*Euterpe precatoria* Mart.	0–4	0	0–2	0–5	0
*Blackseed*	0–1	0–2	1–3	0	0–9
*Couratari multiflora* Eyma	0–7	0–1	0	0–1	0
*Byrsonima stipulacea* Juss	0	0	0	0	1–9
*Loxopterygium sagotii* Hook	0–1	0	0	0–1	0–5
Sandwood	0–1	0	0	0–2	0–2
*Brosimum guianensis* (Aubl.) Huber	0–2	0	0	0–1	0–4
All species	16–20	7–12	1–8	12–21	9–12

Dominant trees classified as those with a mean value >1 tree per plot. Data are trunks >10dbh per 2500 m^2^.

### Forest resource use

During the study period, utilisation was recorded by the sample households for 73 named animal species (plus at least six ethno-species of fish) and 164 named plant species (plus at least 19 unidentified ethno-species). For ease of comparison with other studies these resources are grouped into five use categories: wild animals killed for consumption; wild fruits gathered for consumption; firewood; construction materials; and craft materials (Table 3).

**Table 3:** Number of species used by participants in the North Rupununi, Guyana for each resource use category defined in the study.

Resource category	Number of species used	Comment
Fish	47 plus 6 unidentified ethnospecies	Represents 64% of species record by Eigenmann [66] and 40% of those recorded by Forte [26]
Mammals	10	
Birds	12	Represents 22% of species recorded as ‘generally eaten’ Forte [26]
Reptiles	4 (1 iguana, 2 forest tortoise, 1 water turtle)	
Wild fruit	16 plus 8 unidentified ethnospecies	Forte [26] lists 20 species and a further 40 ethnospecies of which 5 species and 9 ethnospecies were recorded here
Firewood	27 plus 13 unidentified ethnospecies of wild growing trees and 6 domesticated fruit trees	
Construction wood	23 plus 8 unidentified	
Construction material	3 palm species; bark from 1 identified and 1 unidentified tree; stems of 1 identified and 1 unidentified liana	
Craft materials	13 (1 herb, 4 liana, 2 palms and 6 hard wood trees)	

### Patterns of use of animal resources

Of the vertebrate species taken from the wild, fish contributed both the highest number of individuals and the largest proportion (by weight) of wild caught vertebrates consumed (53%). Fish comprised over 98% of the individuals taken; mammals were the most commonly taken terrestrial vertebrates (55%), followed by reptiles (30%) and then birds (15%).

**Figure 5:**
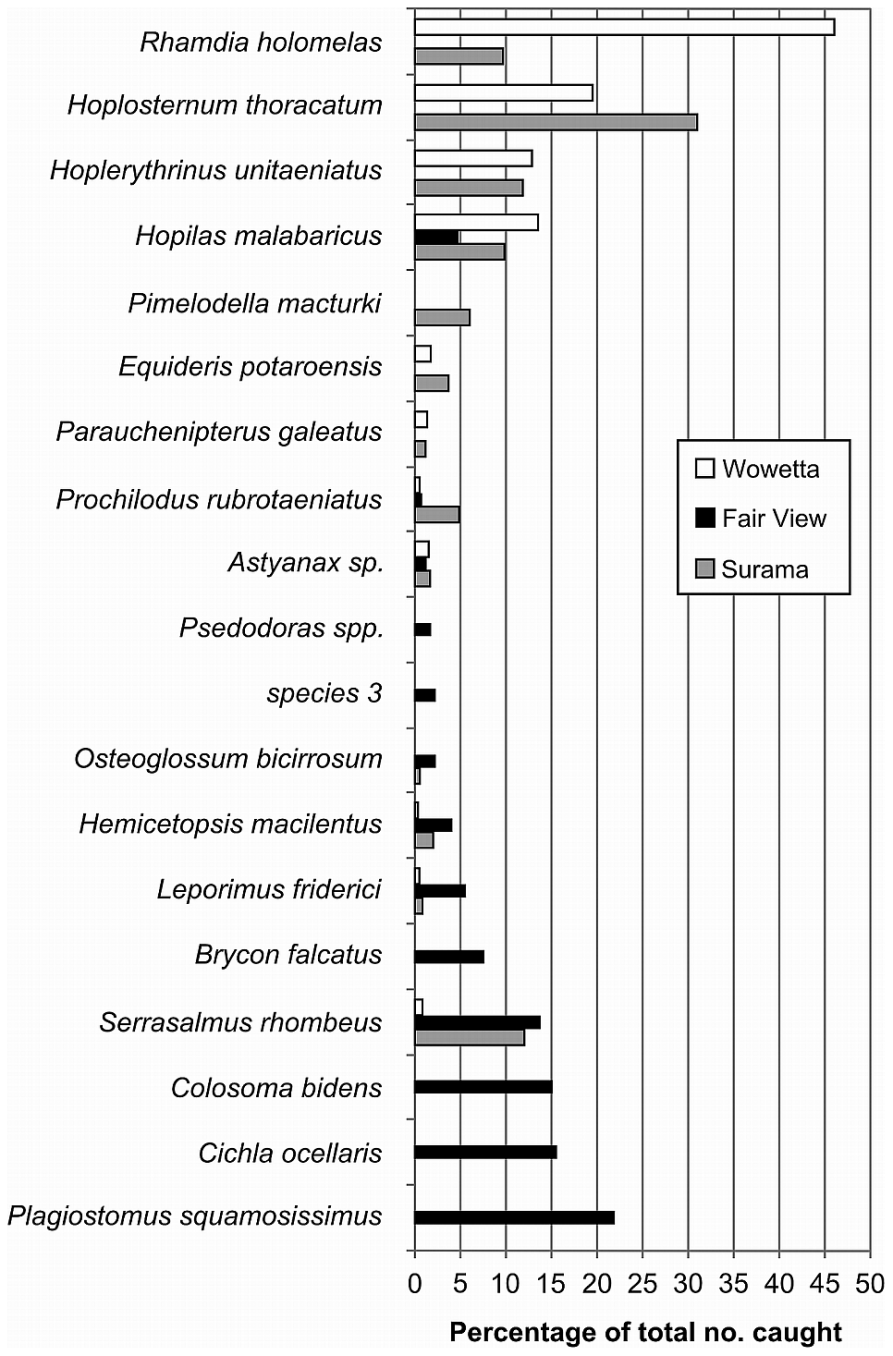
Most commonly caught fish species in each of three villages, North Rupununi Guyana. (Representing 1 percent or more of fish caught in all three communities during the sample year).

The fish species most commonly caught in Surama and Wowetta are those that habitually live in seasonal waterways and smaller creeks and ponds, while those most commonly caught in Fair View are those that generally live in large rivers or associated habitats (Figure 5).

**Figure 6:**
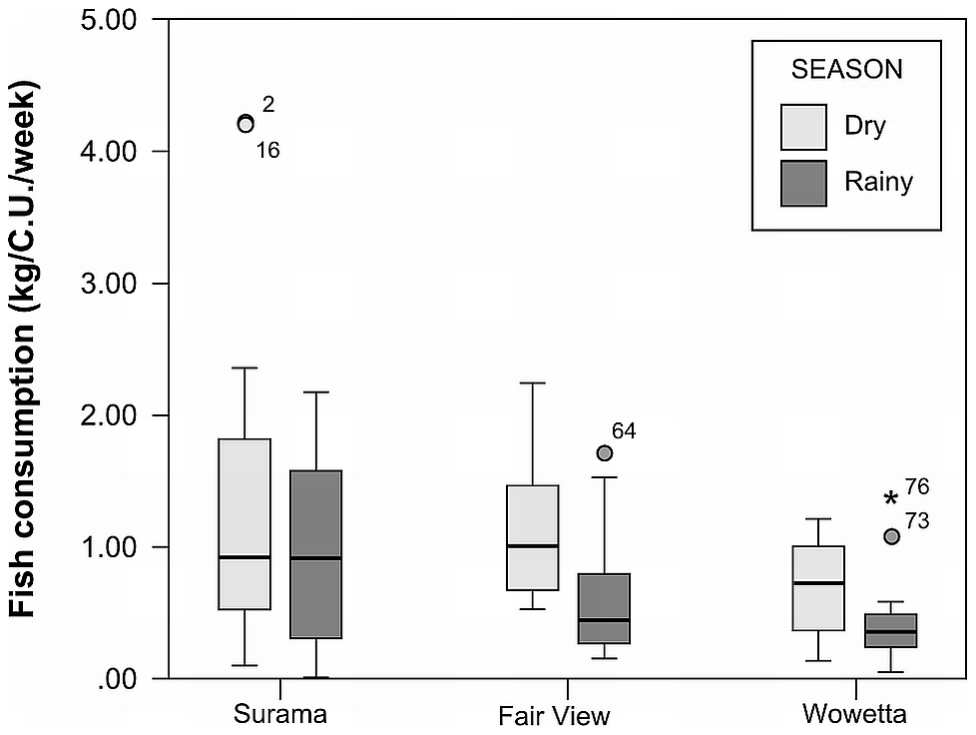
Seasonal and inter-community differences in fish consumption (kg/C.U./week) for three villages, North Rupununi, Guyana.

The seasonal (F_1, 79_ = 25.7; P<0.01) and inter-community (F_2, 79_ = 36.7; P<0.01) differences in quantities of fish consumed were significant (Figure 6), but the relationship with income was non-significant (rs = −0.18, N = 86, p>0.05).

**Figure 7:**
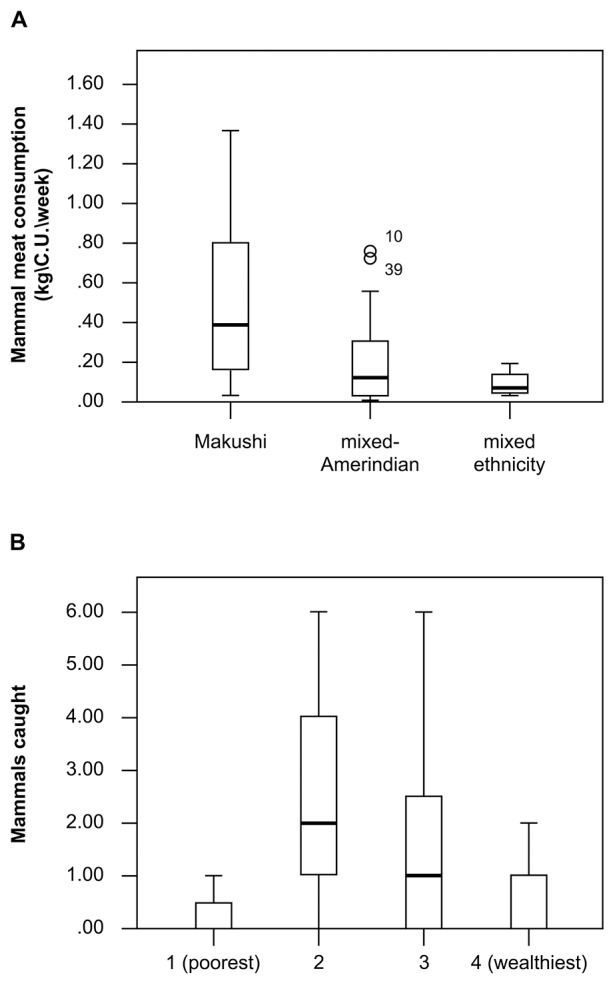
Patterns of animal resource use amongst Amerindian groups, North Rupununi, Guyana. A) Household consumption of mammal meat in the three classes of traditional origins, B) Capture of mammals in four income classes in the North Rupununi, Guyana.

There was a significant difference in mammal meat consumption between ethnicity classes (K-W H = 10.7; df = 2; P = 0.005), (Figure 7), but not in the number of mammals caught (K-W H = 4.4; df = 2; P = 0.111). In contrast, there was a significant difference in the number of mammals caught between income classes (K-W H = 14.1, df = 2, P = 0.003), but not in consumption of mammal meat (K-W H = 2.3; df = 2; P = 0.522). A slight trend of decreasing capture rate with increasing income class was apparent between income class 2 and 4 (Figure 7).

### Patterns of use of plant resources

**Figure 8:**
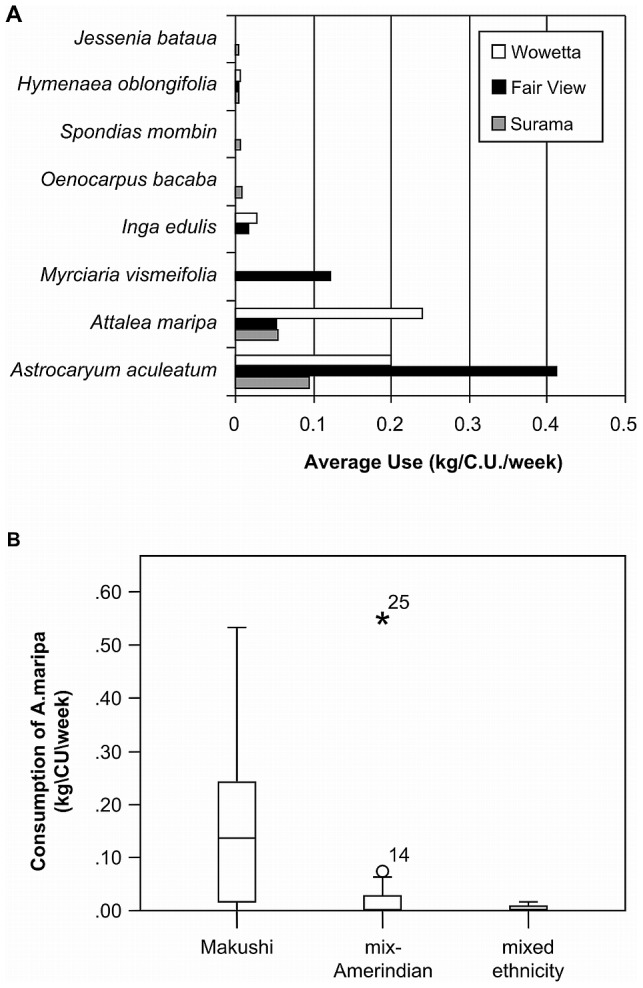
Patterns of wildfruit and palm use by Amerindian groups, North Rupununi, Guyana. A) Wild fruits consumed in three villages (representing more than 1% of total consumption, B) Consumption of *Attalea maripa* by ethnicity.

By weight, 81 percent of the wild fruits consumed were from the two palm species, *Astrocaryum aculiatum* (51%) and *Attalea maripa* (30%). Consumption of *A. maripa* was greatest in households with Makushi traditional origins and least in households with some Mixed-ethnicity, (Figure 8) (K-W H = 14.4; df = 2; P = 0.001). There were no other significant differences in consumption of wild fruits overall or of *A. aculiatum* separately (ANOVA, F  = 1.585; df = 3, 39; P = 0.209).

The most preferred firewood species overall was *Swartzia leiocalycina* Benth, (20% of all firewood collected and used in all three communities). The next most commonly used was *B. spicata*, comprising 19% and only used in Surama and Wowetta. We found no significant differences in species diversity or quantity of firewood used between villages, income groups or ethnic groups. The number of individual timber poles in the 50–150 cm dbh size class (used for house construction) did differ between villages (K-W H = 10.5; df = 2; P = 0.005) with use greatest in Surama, very rare in Wowetta and intermediate in Fair View.

*A. maripa* was the most commonly used palm species. The average number of *A. maripa* leaves used per household was significantly greater in Wowetta than in Fair View and tended towards being significantly greater in Wowetta than in Surama (ANOVA, F = 11.848; df = 2,40; P<0.001; Tukey's post hoc test Wowetta cf. Fair View P<0.001 and Wowetta cf. Surama P<0.019). *A. maripa* leaf use was very low in Fair View where most houses relied upon recycled zinc sheets for roofing. There were no significant differences in use of *A. maripa* leaves for thatching between groups defined according to traditions or income.

The most commonly used species for craft production was *Ischnosiphon arouma (Aubl.)Körn*, used to manufacture traditional household items. The average number of plants used per household differed significantly between communities (K-W H = 77.4; df = 2; P = 0.024), Wowetta using the highest number on average, followed by Surama, while use in Fair View was very rare.

### Forest management

The main forest management activities included agriculture, hunting, fishing, harvesting of wild plant products such as edible fruits, construction materials, craft materials, those with medicinal and/or supernatural purposes, and the cutting of trails to facilitate activities in the forest. All participants very uniformly described the general form of activities and their seasonal cycles.

Agriculture provided a central focus for plant related forest management activities. Farm sites were selected, usually by the male head of household each year, with decisions based on soil qualities, landform and proximity to sites of relatives. Non-inundated, predominantly sandy soils were preferred and 84 percent of farms were in sand or sand-loam soils. The area under cultivation varied between villages from only 9.31 ha in Fairview, where all farms were in close proximity to the village (average distance of 0.39 km), to 29.73 ha in Wowetta and 36.72 ha in Surama, where farms were further away (average distances of 2.94 km and 2.25 km respectively). The average area per household under cultivation was similar for Surama (1.08 ha, S.E. = 0.18) and Wowetta (0.97 ha, S.E. = 0.18) but roughly half that area in Fair View (0.4 ha, S.E. = 0.06).

Opportunism played a key role in the extraction of resources, particularly wild fruits and tortoises, which were usually collected if encountered in the forest but rarely sought out. For example, only 13 percent of fruit collection events were obtained on a trip for which fruit collection was the primary or one of the primary purposes; 65 percent of households did not report seeking out wild fruits at all despite two of the most widely used edible species (the palms *A.maripa* and *A.aculiatum*) being dominant in the “palm” woodland type.

Fishing and hunting trips were made almost entirely to supply household needs rather than primarily to obtain fish or meat to sell (<2%). Men reported that they covered up to 300 km^2^ of the forest during their hunting activities, whilst women usually reported and were observed to visit only more proximate forest locations e.g. the forest on the farm road. A factor universally identified as affecting the decisions to fish or hunt was the amount of mammal meat or fish received as an offering from another household, a practice known as “sharing”. Although a commonly given reason for sharing was to achieve an even distribution of the resource across the community, meat was much more likely to be shared to family than non family (paired sample T-test t = 4.8; df = 42; P<0.001).

### Spatial patterns in extraction of forest resources

The majority of plant resources were collected from the forest around the farm or wild species growing up in the farm itself and it was also an important hunting area. 56 percent (by weight) of firewood was collected from farming grounds or the forest immediately around, 71 percent (by weight) of wild fruits, 31 percent (by number of individuals cut) of ‘round-wood’ (i.e. not cut into boards with a chainsaw), 30 percent of thatching palm leaf, and 28 percent of *I. arouma* for craft. Hunting in the farm provided 11 percent of the mammal and 99 percent of the bird meat collected. Participants often referred to the association between some of the useful species and the secondary forest around the farming areas.

This study recorded no traditionally prohibited areas within the normal ranges of any of the study villages, although specific areas within the range of neighbouring villages were referred to. Hunting trips did tend to visit areas at some distance from the village centres (up to 50 km), as game was scarce in areas close to the population centres. Firewood not collected from the farm areas, was usually obtained from the forest edge as close to the household as possible, although in Wowetta, this still entailed trips of over 2 km from some households.

### Socio cultural differentiation in management strategies

**Figure 9:**
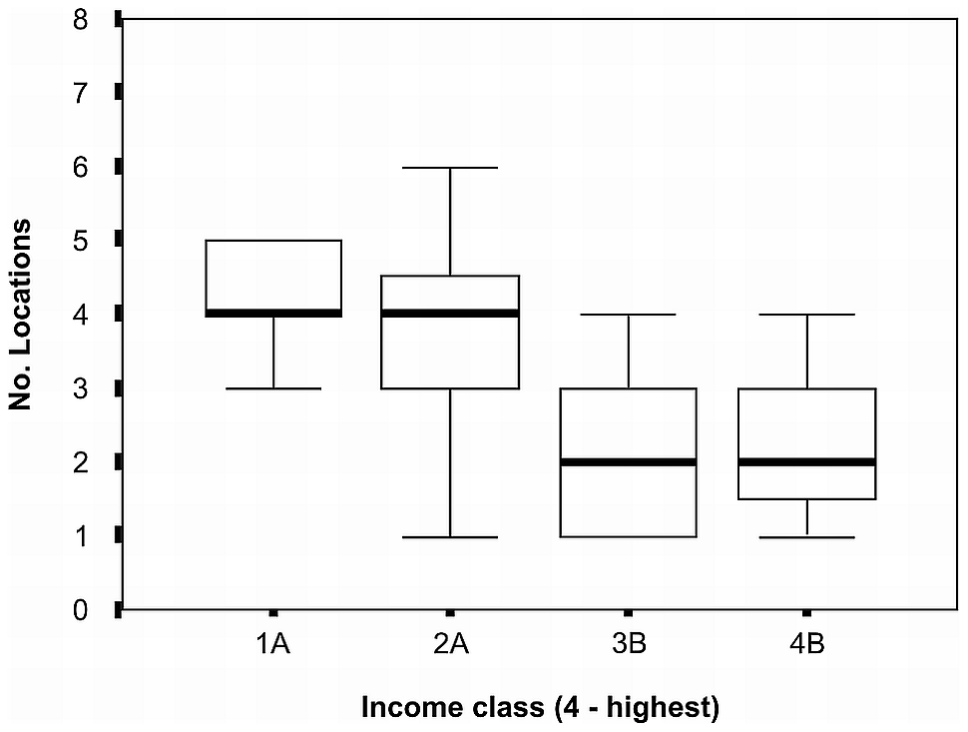
Number of locations for timber collection by income class for Amerindians, North Rupununi, Guyana. Columns with the same letter do not differ according to Tukey's multiple range test.

Participants perceived ethnicity and the related notion of traditionalism (both of which were inversely related to household income), as being the main sources of differences in forest management strategies. The number of different locations from which household timber had been obtained varied with income class (ANOVA, F = 4.282; df = 3, 48; P = 0.009) with a division between the two lowest and the two highest income classes (Figure 9). Households in the lowest income group sourced timber from significantly more locations than those in the two highest income classes. While the majority of hunting was carried out with bow and arrow, three households (all in upper two income groups) in Surama owned guns and relatives from other households regularly borrowed them.

**Figure 10:**
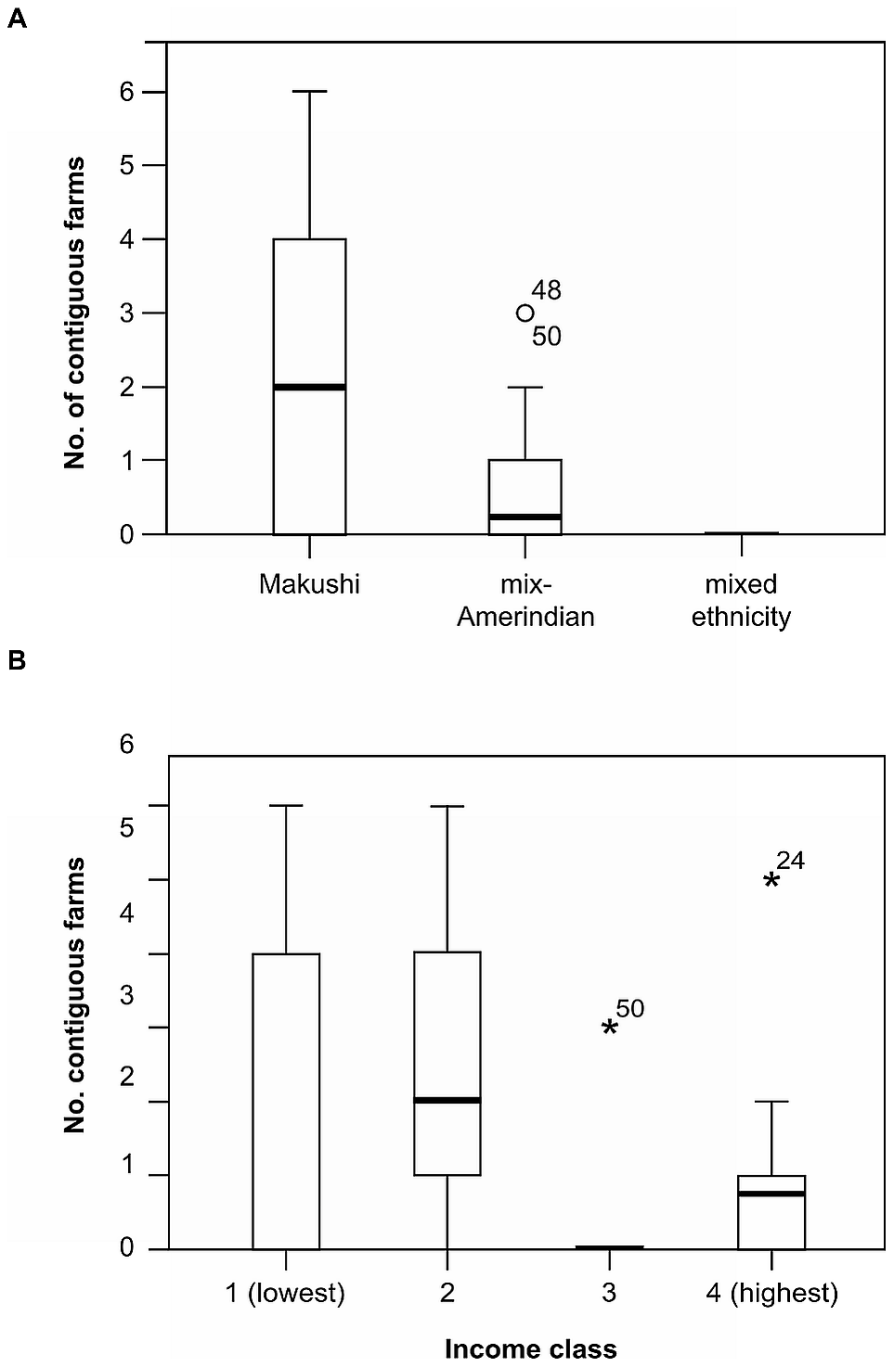
Farm contiguity in the North Rupununi Guyana, by ethnicity and income class. A) Number of contiguous neighbouring farms by ethnicity class of owning household, B) Number of contiguous neighbouring farms by income class of owning household in the North Rupununi, Guyana (Differences significant by Tukey's post hoc, 2>3 *P* = 0.013, 2>4 *P* = 0.028).

The described norm for traditional farming was to have large clusters of farms belonging to households of relatives. Households in the Makushi ethnic group had more contiguous neighbours than those in the Amerindian mixed group, which in turn had more contiguous neighbours than those in the mixed ethnicity group (Figure 10) this difference was significant (K-W H = 12.6; df = 2; P = 0.002). There was also a significant difference in the number of contiguous neighbouring farms between households in income class 2 and the two highest, 3 and 4 (ANOVA, F = 4.952; df = 3,60; P = 0.004) (Figure 10).

## Discussion

### Forest management

The norms of forest resource use and management found in the North Rupununi were similar to those described for many other forest-based Amerindian groups living a subsistence lifestyle (e.g. [49]–[52]). Although early researchers sought to classify Amerindian communities into those that were ‘hunters’, ‘fishers’, ‘gatherers’ or ‘cultivators’, this has long been recognised as an over-simplification [53], [54]. Here we observed that trips to the forest could not be definitively classified as one type of activity, there was an interlinked strategy of activities focussed around supplying household needs. Explicit management activities were rare and although some traditional management activities may have been lost, we suggest that a system of prohibited areas may never have been important in a region where there are large tracts of forest which have not been settled in living memory and resources are not limited. The distances between households and farm sites were very short compared to the estimated 6–10 km average distance between households and farm sites for savannah communities throughout the Rupununi [55]. While distance was certainly a factor in limiting resources extraction from areas around the villages, there were no definite limits except those imposed by difficult to cross topographical features. The forest management observed here is the result of activities that have management effects, but which are incidental to the main agricultural and subsistence objectives of those activities.

### Variation in resource use and forest management strategy with ethnicity and socioeconomic grouping

We found that whilst the use of wild fish is ubiquitous, driven by availability, the amount and type of other sources of dietary protein differed between ethnic groups. In particular, mammal meat consumption and the use of the palm fruits from *A. maripa* were higher in the more traditional Makushi households (Figures 7 and 8). In interviews, participants attributed this pattern of protein consumption to the knowledge and skills base of the people in this group and to ethnically based food preferences. Participants perceived Makushi households as being more “traditional”, a view partly based on their style of house construction, and as more reliant on forest resources. This is the first time such significant differentiation in wild resource use has been recorded at an inter-household level within communities.

Household socioeconomic status also influenced resource use patterns. For example, middle income groups hunted significantly more mammal meat than other groups, but were not consuming more, suggesting they were selling or sharing their catches with members of other income groups. This relationship formed part of a complex pattern of traditional deferred exchange, acting to promote community cohesion and increase equality of access to resources. The patterns of harvesting of construction materials also differed between income groups with more localised extraction and greater number of larger trees being harvested by villagers in the higher income groups. This latter pattern could lead to localised resource depletion and has clear impacts on plant spatial distribution.We found significant within community variations in tree extraction patterns, with lower income groups creating scattered felling sites more akin to tree fall gaps. Such felling gaps have been shown to return to similar levels of diversity and species complement to pristine forest within five years of disturbance in Africa, Bolivia and Borneo [56]–[58]. Higher income groups were more likely to create larger gaps from which greater numbers of trees are extracted which would take longer to recover.

Much of the forest resource use in these communities (with the exception of fish) was concentrated around current and ‘old’ or fallow farm sites. Whilst hunting trips did take participants significant distances from the villages, between 7 and 11 percent of these resources were also derived from the farm sites. Thus these locations form foci for forest management activities. Farms were generally set close to the villages (within 3 km on average), but there were significant differences between villages and between ethnic groups in terms of the spatial patterns of farm distribution and therefore forest resource use, reinforcing the potential for local ”placed based” effects on biodiversity patterns.

At an intermediate landscape scale, forest clearance for farming shows different patterns, with more traditional Makushi households clearing contiguous areas resulting in larger gaps and less traditional households clearing more disparate sites. Evidence from other areas in the tropics suggests smaller more disparate sites may return to similar levels of plant diversity within 50–60 years, but that rare species may not return easily [57], [58].

The spatial pattern of the farms within the North Rupununi forest lies on a scale suggested to increase biodiversity [59], [60] under the intermediate disturbance model [61]. Shifting cultivation and small scale timber extraction have been shown to increase floristic diversity in Northern Guyana [62], also in Africa [63] and Asia [64], marked by an increase in the number of pioneer species. The floristic data here indicate that ex-farm plots do contribute to supporting species that are less common in the surrounding forest therefore increasing diversity reinforcing the conclusions of others about the influence of humans on tropical forest diversity in other regions [8].

We conclude that there are ecologically significant differences in resource use between adjacent villages in the North Rupununi, both within communities (driven by relative affluence) and between community variations (driven by differences in ethnicity). Spatial patterns related to agriculture differed between villages, while differences between timber extraction patterns were driven more by financial status. Our findings that there is a significant role for both income and ethnicity in driving resource use and hence forest biodiversity patterns in the North Rupununi, have implications for models of diversity which underlie conservation planning and can contribute to assessments used in monitoring for REDD+ [65] which has particular importance in Guyana.

## Supporting Information

Appendix S1 **List of plant specimens collected by C. Cabral, University of Roehampton and deposited at the University of Guyana Herbarium.**
(DOCX)
