# Supplementary material for: Variation in Indigenous Forest Resource Use in Central Guyana
Source: PLoS One. 2014 Jul 28;9(7):e102952. doi: 10.1371/journal.pone.0102952 (PMC4113306; doi:10.1371/journal.pone.0102952)
Supplement: Appendix S1 — List of plant specimens collected by C. Cabral, University of Roehampton and deposited at the University of Guyana Herbarium. (DOCX) [file pone.0102952.s001.docx]

**Appendix S1. List of plant specimens collected by C. Cabral, University of Roehampton and deposited at the University of Guyana Herbarium**

**# Local name Family Probable Latin name**

1 Hitcha HALPIGHIACEAE *Byrsonima spicata* (Cav.) DC.

2 Kayambai

3 Mirichi

4 Young-girl EUPHORBIACEAE *Mabea* sp.

6 Trysil LEGUMINOSAE *Pentaclethra macroloba* (Willd.) Kuntze

(MIMOSOIDEAE)

7 Mora LEGUMINOSAE) *Mora excelsa* Benth or M. *gonggrijpii*

(CAESALPINIOIDEAE) (Kleinhoonte) Sandw.

8 Duka ANACARDIACEAE *Tapirira marchandii* Engl.

9 Futi / Pooti / BIGNONIACEAE *Jacaranda copia* (Aublet) D.Don

Fuiti

10 Birdwood

11 Haiawa BURSERACEAE *Protium* spp.

12 Borohuda CHRYSOBALANACEAE *Parinari campestris* Aublet

13 Wild Cherry MYRTACEAE *Eugenia patrisii* Vahl

14 BatSowery

15 Bloodwood GUTTIFERAE *Licania persaudii* or *Vismia* sp.

16 SwampDalli

17 Iron Mary/ LEGUMINOSAE *Clathrotropis paradoxa* Sandw.

Tira (PAPILIONOIDEAE)

18 White Ceder BIGNONIACEAE *Tabebuia* sp.

19 Iteballi VOCHYSIACEAE *Vochysia* sp.

20 Smooth-leaf LECYTHIDACEAE *Eschweilera decolorans* Sandw.,

Kakarali

22 Kereti/ LAURACEAE *Lauraceae* spp.

Silverballi

23 Whitey

24 Aramata (Ar) LEGUMINOSAE *Clathrotropis macrocarpa* Ducke

(PAPILIONOIDEAE)

25 Acourie-broot CHRYSOBALANACEAE *Licania densiflora* Kleinhoonte

(Cr)

26 Cow wood (Cr) MORACEAE *Bagassa guianensis* Aublet Synonym:

*Bagassa tiliifolia* (Hamilton) Benoist

27 Bush Sowery

28 Kanahia

29 Yarriyarri ANNONACEAE *Duguetia* sp.

30 Turu ARECACEAE *Inoenocarpus bataua* Mart. var. *bataua*

31 Cassavamama

32 Congo Pump MORACEAE *Cecropia angulata* I.Bailey,

*C. sciadophylla* C.Martius

33 Blackseed

34 Hill Dalli MYRISTICACEAE *Virola michelii* Heckel

35 Common LECYTHIDACEAE *Eschweilera sagotiana* Miers

black/Kakaralli/Poko/ Kwatru

36 Rough-leaf STERCULIACEAE *Sterculia rugosa* R.Br. Synonym:

Maho *Xylosterculia rugosa* (R.Br.) Kosterm.

37 Wildbanana

38 Locust LEGUMINOSAE *Hymenaea oblongifolia* Huber

(CAESALPINIOIDEAE) *H. courbaril* L.

39 Wallaba (Ar) LEGUMINOSAE poss *Eperua* sp.

(CAESALPINIOIDEAE)

40 Yellow LAURACEAE *Ocotea rhynchophylla* Mez

Silverballi

41 Sand BOMBACACEAE *Catostemma fragrans* Benth.

Baromalli

42 Cinthia

43 Angelina Rock

44 Beads tree LEGUMINOSAE *Ormosia coccinea* (Aublet) B.D.

(PAPILIONOIDEAE) Jackson

45 Clump Wallaba LEGUMINOSAE *Dicymbe altsonii* Sandw.,

(CAESALPINIOIDEAE) *D.corymbosa* Spruce ex Benth.

46 Glasswood

47 Bonewood

48 Haiawaballi BURSERACEAE *Tetragastris altissima* (Ablet) Swart

49 Lapenni / EUPHORBIACEAE *Drypetes variabilis* Uittien

Shibidan

50 Nature Wood

51 Leopard WoodMORACEAE *Brosimum guianensis* (Aubl.) Huber

52 Wamara FABACEAE *Swartzia leiocalycina* Benth.

53 Swamp Fukadi COMBRETACEAE *Terminalia dichotoma* G. Meyer

54 Yarula APOCYNACEAE *Aspidosperma excelsum* Benth,

*A. oblongum* A.DC.

55 Purpleheart LEGUMINOSAE *Peltogyne venosa* (Vahl) Benth.

(CAESALPINIOIDEAE)

56 Lu /Kung] ARECACEAE *Oenocarpus bacaba* Mart. (Synonym

*O.b.* var. *xanthocarp*a Trail)

57 Ite/Quai] ARECACEAE *Mauritia flexuosa* L.f.

58 Lana RUBIACEAE *Genipa americana* L.

59 Simarupa SIMAROUBACEAE *Quassia simarouba* L.f. Synonym:

*Simarouba amrara* Aublet

60 Wild Genip LEGUMINOSAE *Muellera frutescens* (Aublet)

(PAPILIONOIDEAE) Standley

61 Arawana Egg

62 Freijo (Pg) BORAGINACEAE *Cordia alliodora* (Ruíz Lopez & Pavón)

Oken

63 Charcole skin EBENACEAE poss *Diospyros guianensis*

barara

64 Black Kakaralli LECYTHIDACEAE *Eschweilera subglanulosa* Miers

65 Brown LAURACEAE *Licaria cannella* (Meisner) Kosterm

Silverballi

66 Gramma Cherry

67 Burada CHRYSOBALANACEAE *Parinari* sp.or *Excellodendron barbatum*

(Ducke) Prance

68 Kanaria

69 Parikaran

70 Monkey Apple ANNONACEAE *Annona muracita* L.

71 White LAURACEAE *Ocotea canaliculata* (Rich.) Mez

Silverballi

72 Swamp Simaropa

73 Manicole ARECACEAE *Euterpe edulis* Mart.

74 Hill Fukadi COMBRETACEAE *Terminalia amazonia* (J.F. Gmelin) Excell

75 CrabWood MELIACEAE *Carapa guianensis* Ablet

(poss. *C. procera*)

76 Common BOMBACACEAE *Catostemma commune* Sandw.

Baromalli

77 Bush-cow Whitey

78 Baromalli BOMBACACEAE *Catostemma altsonii* Sandw.

79 Brazil Nut LECYTHIDACEAE *Bertholletia excelsa* Humb. & Bonpl.

80 Fukadi (Ar) COMBRETACEAE *Buchenaria fanshawei* Excell & Miguire

81 Kamaka BOMBACACEAE *Bombax globosum* Aublet or

*B. surinamense* Uittien

82 Guava-skin LECYTHIDACEAE *E. alata* A.C.Smith

Kakarali

83 Plum

84 Swamp Parikaran

85 Wild Cashew ANACARDIACEAE *Anacardium giganteum*

W. Hanc. Ex Engl.

86 Serebedan FABACEAE *Swartzi oblanceolata* Sandw.

87 Hill Cork wood LEGUMINOSAE *Pterocarpus rohrii* Vohl

(PAPILIONOIDEAE)

88 Counter CRYSOBALANACEAE *Licania alba* (Bernoulli) Cuatr.,

Synonym: *L. venosa* Rusby

89 Bartaballi SAPOTACEAE *Ecclinusa guianensis* Eyma

90 Black ANNONACEAE *Guatterria* sp.

yarriyarri

91 Boba ARECACEAE *Iriatea exorrhiza* C.Martius

92 Bullet Wood SAPOTACEAE *Manilkara bidentata* (A.DC.) Chev.

93 Hububali ANACARDIACEAE *Loxopterygium sagotii* Hook.f.

94 Kuyuru ARECACEAE *Astrocaryum aculiatum* G.Meyer

(Synonym *A. tucuma* C.Martius)

95 Red Cedar MELIACEAE *Cedrela odorata* L.

96 Washiba BIGNONIACEAE *Tabebuia* sp. nov.

97 Fine-leaf LECYTHIDACEAE *Eschweilera wachenheimii* (Benoist)

kakarali Sandw.

98 Kabukalli CELASTRACEAE *Goupia glabra* Aublet,

NB: sometimes place in separate family: Goupiaceae

99 Sand wood

100 Wamaradang

101 Smooth-leaf STERCULIACEAE *Sterculia pruriens* (Aublet.) Schumann

Maho

102 Kokerite ARECACEAE *Attalea maripa*

103 Kauta/ CHRYSOBALANACEAE *Licania laxiflora* Fritsch

Counta

104 Wadara LECYTHIDACEAE *Couratari* sp.

105 Greenheart LAURACEAE *Chlorocardium rodiei* (Schomb.)

Rohwer, Richter & van der Werff,

106 Tonka Bean LEGUMINOSAE *Dipteryx* spp.or *Taralea*

(PAPILIONOIDEAE) *oppositifolia* Aublet,
